# What Is the Efficacy of Intra-articular Injections in the Treatment of Ankle Osteoarthritis? A Systematic Review

**DOI:** 10.1097/CORR.0000000000002624

**Published:** 2023-04-11

**Authors:** Liam D. A. Paget, Milo J. Mokkenstorm, Johannes L. Tol, Gino M. M. J. Kerkhoffs, Gustaaf Reurink

**Affiliations:** 1 Amsterdam UMC, University of Amsterdam, Department of Orthopedic Surgery and Sports Medicine, Amsterdam Movement Sciences, Amsterdam, the Netherlands; 2 Academic Center for Evidence-based Sports Medicine, Amsterdam, the Netherlands; 3 Amsterdam Collaboration for Health and Safety in Sports, AMC/VUmc International Olympic Committee Research Center, Amsterdam, the Netherlands; 4 Aspetar, Orthopedic and Sports Medicine Hospital, Doha, Qatar; 5 The Sport Physician Group, Department of Sports Medicine, Onze Lieve Vrouwe Gasthuis, Amsterdam, the Netherlands

## Abstract

**Background:**

Ankle osteoarthritis (OA) is painful and can impact a patient’s physical and mental quality of life. Although intra-articular injections are commonly used to alleviate symptoms, there is conflicting evidence concerning their efficacy. Therefore, an updated systematic review would be informative.

**Question/purpose:**

In this systematic review, we asked: Are there clinically important benefits or harms associated with the use of intra-articular injections in the treatment of ankle OA?

**Methods:**

We used PubMed, Embase, and the Cochrane Library to search for randomized controlled trials on intra-articular injections for the treatment of ankle OA in June 2021, and updated the search in January 2022; eligible dates were from the date of inception of each database through January 2022. Reference lists of eligible studies and previous reviews were manually screened. Two reviewers independently assessed studies for eligibility. We included seven studies. Three compared hyaluronic acid (HA) with saline, one compared HA with exercise, one compared four different regimens of HA [34], one compared platelet-rich plasma (PRP) with saline, and one compared botulinum toxin Type A (BoNT-A) with HA. A total of 340 patients were included: 141 in the HA arms, 48 in the PRP arm, 38 in the BoNT-A arm, and 113 in the saline arms. Across all studies, the mean age was 52 ± 21 years, and 35% were women (119 of 340 patients). Methodologic quality was assessed using the Cochrane Risk of Bias 2.0 tool. Of the included studies, the risk of bias was low in two studies, presented some concerns in one study, and was high in four studies. According to the Grading of Recommendations Assessment, Development, and Evaluation methodology, the level of evidence was very low for HA, moderate for PRP, and very low for BoNT-A. The level of heterogeneity was high, and we opted to perform a systematic review rather than a meta-analysis. A clinically relevant difference was based on whether the between-group difference surpassed the cutoff point determined as the minimum clinically important difference.

**Results:**

No clinically relevant differences were found among HA, PRP, and BoNT-A and their control groups at 3, 6, or 12 months. No studies reported any serious adverse events in any treatment group.

**Conclusion:**

Given the lack of observed efficacy in this systematic review, these treatments should not be used in practice until or unless future high-quality studies find evidence of efficacy.

**Level of Evidence:**

Level III, therapeutic study.

## Introduction

The estimated incidence of ankle osteoarthritis (OA) is 3.4%, 70% to 78% of which is posttraumatic [23, 24, 29]. In professional former football and rugby players, ankle OA has been associated with previous ankle injuries, and the estimated incidence of ankle OA is higher (9.2% and 4.6%, respectively) in these athletes than in the general population [17]. The quality of life of patients with ankle OA may be impacted substantially, and the level of diminished quality of life from ankle arthritis has been shown to be comparable to that reported for hip OA, end-stage kidney disease, and congestive heart failure [9, 19, 24]. Early attempts at ankle arthroplasty had poor results, but the design of contemporary third-generation implants has led to considerably improved outcomes such as preserved motion, restoration of normal gait, and improved survivorship [32]. However, complications after ankle arthroplasty remain more common than after knee or hip arthroplasty, and because of this, ankle arthrodesis remains the preferred treatment for most patients [10, 28]. Because patients with ankle OA tend to be younger and more active than patients with knee and hip OA, their longer projected lifespan is impacted more severely by a reduced quality of life or substantial functional limitations after ankle arthrodesis. Owing to these concerns, nonsurgical approaches are a mainstay of the treatment of patients with ankle OA, and intra-articular injections are commonly used to alleviate ankle OA symptoms.

Previous systematic reviews on the efficacy of intra-articular injections for ankle OA have had methodologic issues, including pooling the results of studies with a high risk of bias and inferior methods for synthesizing evidence [2, 31]. Furthermore, since the publication of those previous reviews, one new trial has been published on intra-articular interventions for ankle OA [18]. There is therefore a need for an updated synthesis of the best available evidence.

With this in mind, we performed a systematic review in which we asked: Are there clinically important benefits or harms associated with the use of intra-articular injections in the treatment of ankle arthritis?

## Patients and Methods

The study protocol was prospectively registered in the International Prospective Register of Systematic Reviews with registration number CRD42021254200. This study used the Preferred Reporting Items for Systematic Reviews and Meta-analyses statement as a guideline.

### Eligibility Criteria

Randomized controlled trials (RCTs) evaluating intra-articular injection therapy for ankle OA were eligible for this review. We excluded articles written in languages other than English, Dutch, or German and studies not conducted in humans.

### Information Sources and Search Strategy

**Fig. 1:**
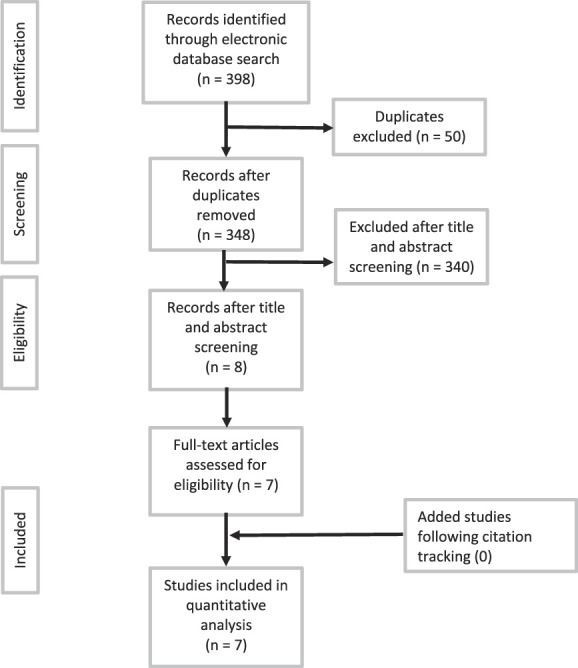
This flow diagram shows the results of the search.

We searched the databases of Medline (PubMed), Embase (Ovid), and the Cochrane Library from the date of inception of each database through January 2022. The main keywords in our searches were ankle joint, osteoarthritis, and intra-articular injections. We tracked citations by manually screening the reference lists of eligible studies and previous reviews as well as conference proceedings. The search revealed 398 studies. After duplicates were removed, 348 records remained. Eight relevant studies remained after title and abstract screening. After a full-text review, seven studies [5, 6, 13, 18, 22, 27, 34] met the inclusion criteria. No additional studies were found through citation tracking or conference proceedings (Fig. 1), and we did not consider articles on preprint servers.

### Selection Process

Two reviewers (LDAP and MJM) independently assessed studies for eligibility. These two reviewers screened titles and abstracts and obtained relevant articles. Those same reviewers independently read the full text of all relevant articles. Eligible articles were included. A third reviewer (GR) resolved any difference in opinion where no consensus could be reached.

### Study Characteristics

Seven studies were included, (Table 1). Hyaluronic acid (HA), platelet-rich plasma (PRP), botulinum toxin Type A (BoNT-A), and saline were reported as the intra-articular interventions in the included RCTs. The studies included three comparing HA with saline [5, 6, 22], one comparing HA with exercise [13], one comparing four different regimens of HA [34], one comparing PRP with saline [18], and one comparing BoNT-A with HA [27]. In total, 15 patient groups were identified with nine arms assessing HA, one arm assessing PRP, four arms assessing saline, and one arm assessing BoNT-A. Across all studies, the mean age was 52 ± 21 years, and 35% were women (119 of 340 patients). A total of 340 patients with ankle OA were included: 141 in the HA arms, 48 in the PRP arm, 38 in the BoNT-A arm, and 113 in the saline arms. Of the six studies with HA as an intervention (three studies versus saline [5, 6, 22], one versus other HA regimens [34], one versus a progressive ankle exercise program [13], and one versus BoNT-A [27]), two studies had a follow-up of 3 months [5, 6], three studies had a follow-up of 6 months [5, 22, 34], and one study had a follow-up of 12 months [13].

**Table 1.** Characteristics and outcomes of included studies

Study	Intervention	Product (manufacturer)	Injection regimen (guidance)	Age in years, mean ± SD (or range)	Gender, % men	Symptom duration in years, mean ± SD (or range)	Follow-up length in weeks	Number of ± SD patients lost to follow-up	Manufacturer funding, yes/no	Main results
HA versus saline										
DeGroot et al. [6] (2012) (n = 64)	HA (n = 39)	Supartz® (Seikagaku) Low molecular weight: 620 to 1170 kDa	One injection under 2.5-mL fluoroscopic guidance	54.1 ± 14.5	61.5%		12	4	No	No between-group differences 3 months: AOS p = 0.14^a^; effect size = 0.4, AOFAS p = 0.90^a^; effect size = 0.4, VAS p = 0.31^a^: effect size = 0.3
	Saline (n = 37)			61.9 ± 14.1	55.5%			4		
Cohen et al. [5] (2008) (n = 28)	HA (n = 15)	Hyalgan® (Fidia Farmaceutici S.p.A.) Low molecular weight 500-730 kDa	Five 1-mL injections with weeklyfluoroscopic guidance	56.2 ± 14.1	93%		26	1	Yes	No clinically relevant between-group differences 3 months: AOS p = 0.04^a^; effect size = 1.0, WOMAC p = 0.03^a^; effect size = 1.0 6 months: AOS p = 0.14^a^; effect size = 0.7, WOMAC p = 0.12^a^; effect size = 0.7
	Saline (n = 13)			43.4 ± 14.9	85%			1		
Salk et al. [22] (2006) (n = 17)	HA (n = 9)	Hyalgan® Low molecular weight 500 to 730 kDa	5 injections of 1 mL (weekly)No guidance	57.8 ± 1.8	56%		26	1	Yes	No between-group differences 6 months: AOS p = 0.32^a^; effect size^b^, WOMAC p > 0.05^a^; effect size^b^
	Saline (n = 8)			60.0 ± 13.9	63%			2		
**HA versus HA**										
Witteveen et al. [34] (2010) (n = 26)	HA (n = 7)	Orthovisc® (Anika Therapeutics) Moderate molecular weight: 1000-2900 kDa	One injection of 1 mL No guidance	31 (26 to 28)	57%		27	2	No	
	HA (n = 7)		One injection of 2 mL No guidance	47 (33 to 63)	43%			4		
	HA (n = 6)		One injection of 3 mL No guidance	51 (39 to 71)	83%			1		
	HA (n = 6)		Three injections of 1 mL (weekly) No guidance	40 (21 to 63)	100%			2		
HA versus progressive exercise program										
Karatosun et al. [13] (2008)	HA (n = 15)	Adant® (Meiji Seika Pharma Co) Low molecular weight: 600 to 1200 kDa	Three injections of 2.5 mg (weekly) No guidance	52.1 ± 11.3	60%		52	0	Unclear	No between-group differences 12 months: AOFAS p > 0.05^a^; effect size^b^, VAS p > 0.05^a^; effect size^b^
	PE (n = 15)		Home-based PE program + four in-hospital training sessions	25.1 ± 12.1	80%			0		
**PRP versus saline**										
Paget et al. [18] (2021) (n = 100)	PRP (n = 48)	ACP Double Syringe System® (Arthrex)	Two injections of 2 mL (6-week interval) ultrasound guidance	54.8 ± 13.3	54%	5 (2 to 8)	26	0	No	No between-group differences 3 months: AOS -3 (-12 to 6) p = 0.47; effect size^b^, VAS 1 (-8 to 11) p = 0.82; effect size^b^ 6 months: AOS 0 (-8 to 9) p = 0.90; effect size^b^, AOFAS -1 (-6 to 3) p = 0.56; effect size^b^, VAS -2 (-12 to 8) p = 0.71; effect size^b^
	Saline (n = 52)			56.4 ± 14.4	56%	8 (3 to 14)		0		
BoNT-A versus HA with rehabilitation exercise										
Sun et al. [27] (2014) (n = 75)	BoNT-A (n = 38)	100-unit of BoNT-A (Allergan, Inc) in 2 mL saline Hyalgan® (Fidia Farmaceutici S.p.A.) Low molecular weight: 500 to 730 kDa	One injection of 2 mL No guidance	49.5 ± 10.9	62.5%	5.6 ± 3.7	26	3	No	
	HA + EX (n = 37)			50.6 ± 10.3	61.2%	5.3 ± 4.0		2		

A negative value shows superiority in favor of the first-named intervention. The AOS ranges from 0 to 100, with an MCID of 28 points; the AOFAS score ranges from 0 to 100, with an estimated MCID of 12 points; the VAS score has an estimated MCID of 20 points; the WOMAC has an estimated MCID of 20 points.

a Apart from the p value, no further data were available; the effect size had the following cutoff points: < 0.2 = small effect; 0.2 to < 0.5 = medium effect; and > 0.8 = large effect.

b It was not possible to calculate effect size. HA = hyaluronic acid; PE = progressive exercise program; ACP = autologous conditioned plasma; PRP = platelet-rich plasma; BoNT-A = botulinum toxin Type A; EX = exercise; MCID = minimum clinically important difference; AOS = Ankle Osteoarthritis Scale; AOFAS = American Orthopaedic Foot and Ankle Society score.

In these studies, low–molecular weight and medium–molecular weight HA was used. Ultrasound-guided injections were performed in three studies [5, 6, 18]. Injection schedules varied from single injections to five injections with varying dosages. The trial follow-up durations ranged from 3 to 12 months. The most frequently used scores were the VAS pain score and the Ankle Osteoarthritis Scale, followed by the American Orthopaedic Foot and Ankle score and WOMAC (Table 1).

### Data Collection Process

One reviewer (MJM) extracted data using a standardized template. Information extracted from each trial included the characteristics of trial participants (including age, gender, years since diagnosis, history of trauma, and radiologic grade) and the trial’s inclusion and exclusion criteria; type of interventions, controls, and regimen (dose, frequency, injection interval); type of outcome measure and value; safety data (including the incidence of adverse events and serious adverse events and discontinuations because of adverse events); and funding.

### Outcomes

We determined that outcome was the between-group difference in any patient-reported outcome measure score for ankle pain or function such as (but not limited to) the American Orthopaedic Foot and Ankle Society score (range 0 to 100 points, with higher scores indicating less pain and better function), Ankle Osteoarthritis Scale (range 0 to 100 points, with higher scores indicating more symptoms), VAS (range 0 to 10 mm, with higher scores indicating more pain), and Foot and Ankle Outcome Score (all scales range from 0 to 100 points, with higher scores indicating less symptoms) at 3, 6, and 12 months as well as any radiologically determined cartilage response (such as increase in radiologic cartilage thickness), side effects, or safety outcomes reported. The clinically relevant difference was based on the between-group mean difference relative to the (estimated) minimum clinically important difference (MCID) of the patient-reported outcome measure used to measure ankle OA. The MCID has not yet been determined for multiple patient-reported outcome measures, but was estimated to be 20 points for the VAS [20], 12 points for the American Orthopaedic Foot and Ankle Society score [18], and 20 points for the WOMAC score [20]. The reported MCID for the Ankle Osteoarthritis Scale was 28 points [3], and for the subscales of the Foot and Ankle Outcome Score (five scales: pain, symptoms, quality of life, activity of daily living, and sport and recreation), it was 15, 7, 18, 23, and 21, respectively [25, 30]. Effect sizes were calculated (if there were sufficient data) as follows: (mean pretreatment – mean posttreatment) / pooled standard deviation. Effect size was defined as small (0.2 to < 0.5), medium (0.5 to < 0.8), or large (≥ 0.8) [4].

### Risk of Bias

Two reviewers (LDAP and MJM) independently assessed the risk of bias in the included studies using the Cochrane Risk of Bias tool 2.0 [26]. If there was a difference in opinion, the two reviewers reached a consensus. Otherwise, the independent opinion of a third reviewer (GR) was used to decide. When one of the primary risk-of-bias assessors was involved as a coauthor of one of the included studies, the noncoauthor risk-of-bias assessor (MJM) evaluated the articles to avoid a conflict of interest over these studies. His assessment was seconded by an independent reviewer (JD, not a study author).

**Fig. 2:**
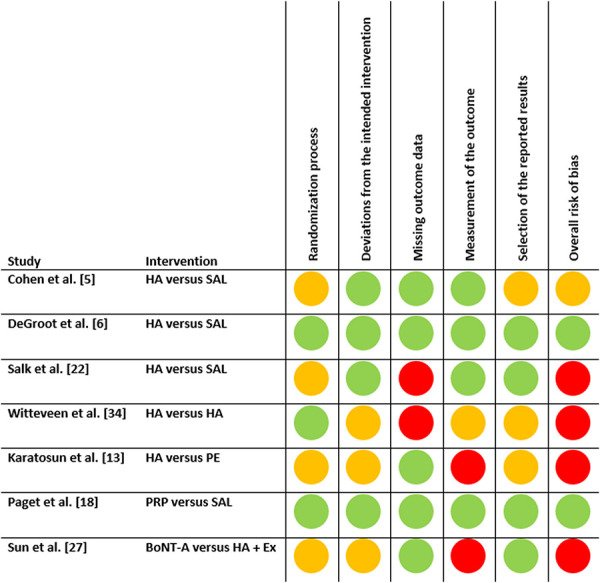
This figure demonstrates the Cochrane Risk of Bias assessment of the included RCTs. BoNT-A = botulinum toxin-A; HA = hyaluronic acid; PRP = platelet-rich plasma; PE = progressive exercise; SAL = saline; EX = rehabilitation exercises. A color image accompanies the online version of this article.

The risk of bias of the included RCTs was assessed as low in two studies, presented some concerns in one study, and was high in four studies (Fig. 2).

The level of evidence of each outcome was graded as high, moderate, low, or very low according to the Grading of Recommendations Assessment, Development, and Evaluation (GRADE) criteria [21]. Because all included studies were RCTs, they started at a high level of evidence. The level of evidence for an outcome was downgraded one or two levels (depending on how serious the quality of evidence was impacted) for each of the five criteria. Very low was the lowest level of evidence, regardless of whether the level could have been downgraded more. This means that for an intervention to have a very low level of evidence (the lowest level of evidence), it must be downgraded at least three levels. Should an intervention be downgraded five levels, it will still be graded as having a very low level of evidence and would seem (according to the GRADE criteria) to have an equally low level of evidence as an intervention that has been downgraded three levels.

The five criteria were risk of bias; inconsistency in the results of published data; indirectness, that is, how well the available data answer the review’s question (for example, when a comparison group is not an ideal control group); imprecision, in which we assessed whether there was enough information (for instance, the rule of thumb is that the total number of participants for an outcome should be more than 400); and publication bias. As with the risk of bias assessment, if one of the GRADE assessors was a coauthor of one of the included studies, it was assessed by assessors not involved in that study (MJM or JD). Any discrepancy was resolved by a third assessor (GR).

The level of evidence for HA was very low (Table 2). The level of evidence was downgraded a total of five levels, two levels for a high risk of bias among most studies, one level for inconsistency in results, one level for indirectness because the small number of studies used different regimens and HA products, and one level for imprecision because there were fewer than 400 participants in total. No publication bias was detected. The level of evidence could not be downgraded lower than a very low level of evidence. The level of evidence for PRP was moderate. Because there is only one RCT on the efficacy of PRP [18], it was downgraded one level for imprecision because the total number participants was fewer than 400 (Table 2).

**Table 2.** GRADE evaluation of the included interventions

Intervention	Risk of bias	Inconsistency of results of published data	Indirectness^a^	Imprecision^b^	Publication bias	Total level of evidence
Hyaluronic acid	-2	-1	-1	-1	0	Very low
Platelet-rich plasma	0	0	0	-1	0	Moderate
Botulinum toxin-A	-2	0	0	-1	0	Very low

Each intervention starts off at a high level of evidence and is potentially downgraded one or two levels from high to moderate, low, and very low, with very low being the lowest level possible.

^a^How well the available data answer the review question (for example, when a comparison group is not an ideal control group).

^b^Is there enough information (for instance, the rule of thumb is that the total number of participants for an outcome should be more than 400).

The level of evidence for BoNT-A was very low. The level of evidence was downgraded a total of three levels, two levels because of serious risk of bias concerns in the one study available and one level for imprecision because the total number of participants was fewer than 400 (Table 2).

### Statistical Analysis

We planned to perform a meta-analysis if at least two studies had the same or similar interventions and comparators and the studies had a low risk of bias [12]. In the event the included studies were not suited for a meta-analysis, we planned to perform a best-evidence synthesis. There was only one study concerning PRP and BoNT-A. Five studies compared HA and saline [5, 6, 13, 22, 34]. However, these studies had different follow-up timepoints and outcome measures. Only three [5, 6, 22] could be compared at 3 months; only one had a low risk of bias, and two [5, 22] could be compared at 6 months, none of which had a low risk of bias. To avoid compounding errors related to the risk of bias, we did not perform a meta-analysis.

## Results

### Benefits of Injectable Products for Ankle OA

No clinically relevant differences were found in any of the studies assessing the efficacy of HA (Table 1) [5, 6, 13, 22, 34].

Paget et al. [18] evaluated the efficacy of two PRP injections versus saline injections and found no between-group differences for any of the reported patient-reported outcome measures at any timepoint (Table 1). Sun et al. [27] evaluated the efficacy of BoNT-A and found no between-group differences for any of the patient-reported outcome measures at any timepoint (Table 1). No clinically relevant differences were found in any of the patient-reported outcome measures at any timepoint (Table 3).

**Table 3.** Interventions and outcomes of RCTs

Intervention	Number of studies	Outcome	Effect			Level of evidence
			12 weeks	26 weeks	52 weeks	
Hyaluronic acid	5	AOFAS AOS VAS WOMAC	x x x x	NR x x x	x NR x NR	Very low
Platelet-rich plasma	1	AOFAS AOS VAS	x x x	x x x	x x x	Moderate
Botulin toxin type-A	1	AOFAS AOS VAS	x x x	x x x	NR NR NR	Very low

“x” indicates there was no clinically important differences between intervention and control. NR = not reported; AOFAS = American Orthopaedic Foot and Ankle Society score; AOS = Ankle Osteoarthritis Scale.

### Harms

No severe or systemic adverse events were reported during the postinjection follow-up period in any treatment group. The most frequently reported adverse events were mild pain and local swelling at the injection site, the proportions of which ranged between 6% and 29% in three studies (no between-group differences) [22, 27, 34]. Witteveen et al. [34] reported pain and swelling in 27% of patients (seven of 27 patients) across all arms. All reported adverse events resolved without medical intervention.

## Discussion

Ankle OA substantially impairs a patient’s mental and physical quality of life. Intra-articular injections are commonly used to alleviate symptoms, but evidence of their efficacy is conflicting. We therefore performed an updated systematic review of the best-available evidence. No clinically relevant differences were found among HA, PRP, and BoNT-A and their control groups in the treatment of ankle OA. After using the GRADE tool, the level of evidence was very low for HA, moderate for PRP, and very low for BoNT-A. No studies reported any serious adverse events in any treatment group. There is currently insufficient evidence to justify the use of any type of intra-articular injection in ankle OA treatment.

### Limitations

The available evidence is limited by a lack of high-quality, placebo-controlled trials, and most studies had small patient groups. Only two RCTs that assessed PRP and HA were classified as having a low risk of bias, with both reporting no statistical differences between the intervention and placebo (saline) groups. The low number of patients, low methodologic quality, and lack of a placebo control group may have resulted in an overestimation of the apparent benefits of treatment. Given the lack of clinically important benefits found in our systematic review, this limitation seems especially important, because it is even less likely that these treatments are worthwhile. The heterogeneity of the included studies regarding important matters such as follow-up period, outcomes used, and intervention regimens limit a meaningful interpretation of the available evidence. The low number of studies and participants, as well as heterogeneity, limits the possibility of performing a subgroup analysis on the efficacy of interventions during different stages of the disease or a meta-analysis. Additionally, studies about intra-articular corticosteroid injections, which are frequently used in practice, or emerging regenerative interventions such as stem cells are extremely limited. Consequently, results are extrapolated from studies concerning knee OA, which is a different joint both biomechanically and biologically. Only one study assessed the efficacy of methylprednisolone in 12 patients (24 ankles with OA) and found an improvement in the Foot and Ankle Outcome Score with 6 months of follow-up [33]. However, the lack of randomization, blinding, a control group, and small sample mean that the quality of evidence of that study was poor. In knee OA, corticosteroid injections are considered controversial because of contradictory short-term and long-term results and because their use may worsen cartilage degeneration [15, 16, 35]. Although mesenchymal stem cell therapy has been used in the knee, a recent meta-analysis found that six of nine included trials on the topic had a high risk of bias [11], which means that the results likely appear better in trials than they would in real-world practice. So far, to our knowledge, the only study on the topic in patients with ankle arthritis included only six patients [7]; more research is needed before conclusions can be drawn regarding the utility, if any, of this approach. Additionally, because no analysis was done based on gender, we cannot assume these findings apply equally to men and women. In four studies, the men-to-women ratio was between 50% to 65% in favor of men; in the three other studies, men represented up to 80% to 93% of the intervention arms (Table 1). However, because there is little overall evidence of efficacy, these treatments should not be used in men or women.

### Benefits and Harms of Injectable Products for Ankle OA

#### HA

There is no evidence to support the use of HA as a treatment for ankle OA. Previous reviews reported promising results for HA. However, of the five studies we evaluated in this systematic review, only one study had a low risk of bias and only one study found a statistical benefit, but none found clinically relevant effect-size differences for the Ankle Osteoarthritis Scale and WOMAC scores at 12 weeks [2, 31], because none of the between-group differences were greater than the relevant MCIDs. No new RCTs have been published on the efficacy of intra-articular HA injections since the publication of the previous reviews on injection therapy for ankle OA [2, 31]. In a previous review, two studies with saline as a control group were pooled at 26 weeks, reporting a mean difference of 18 points on the Ankle Osteoarthritis Scale (range 0 to 100) at 26 weeks [2]. However, we have a methodologic concern about this analysis, because these two studies with risk of bias concerns should not have been pooled [5, 22]. Pooling heterogeneous studies that are at moderate or high risk of bias compounds errors and may produce misleading conclusions that may be interpreted as having more credibility [12]. Two studies with saline as a control group were pooled at 12 weeks and reported a mean difference of 1 point on the Ankle Osteoarthritis Scale. The study that favored HA had a high risk of bias [5], in contrast to the study that favored saline, which had a low risk of bias. In general, studies that are at high risk of bias will tend to overestimate the benefits of the treatment being studied. Overall, despite common use, there is no robust evidence that HA works in the treatment of ankle OA. Pooling and compounding errors resulted in a mean difference of only 18 points on the Ankle Osteoarthritis Scale, which is lower than the reported MCID of 28 points [3], suggesting that any benefit with the use of those products is so small that it might not be considered important by patients, if they even perceive the difference. HA therefore should not be used in practice until or unless high-quality studies find evidence of efficacy [14].

#### PRP

No between-group difference in ankle symptoms and function beyond 26 weeks was seen in the first and only RCT on the efficacy of PRP in the treatment of ankle OA of which we are aware [18]. PRP is a high concentrate of platelets achieved through centrifugation of the patient’s blood after venipuncture [8, 36]. The platelets are thought to release growth factors and cytokines that modulate the intra-articular environment, facilitating an anabolic effect and reducing inflammation [8, 36]. However, the no-difference finding suggests that PRP should not be used in practice for this indication.

#### BoNT-A

BoNT-A is thought to inhibit neurotransmitters such as acetylcholine, thereby reducing peripheral sensitization and indirectly reducing central sensitization [1]. In ankle OA, only one nonblinded RCT was available; at 26 weeks, this treatment was not shown to be as effective as HA injections [27], which our systematic review found was not effective, so until or unless high-quality randomized trials demonstrate otherwise, we recommend against the use of this product as well.

### Conclusion

This systematic review found insufficient evidence to justify the use of intra-articular injections of HA, PRP, or BoNT-A for the treatment of patients with ankle OA. Because of this, we recommend against their use in practice until or unless future high-quality studies find evidence of efficacy. Future studies should be randomized, blinded, well-powered, and placebo controlled.
